# Cellular and micro-environmental responses influencing the antitumor activity of *all-trans* retinoic acid in breast cancer

**DOI:** 10.1186/s12964-024-01492-2

**Published:** 2024-02-15

**Authors:** Maria Azzurra Caricasulo, Adriana Zanetti, Mineko Terao, Enrico Garattini, Gabriela Paroni

**Affiliations:** Department of Biochemistry and Molecular Pharmacology, Laboratory of Molecular Biology, Istituto di Ricerche Farmacologiche Mario Negri IRCCS, Via Mario Negri, 2, Milan, 20156 Italy

**Keywords:** Retinoic acid, Breast cancer, Tumor micro-environment, Differentiation therapy

## Abstract

*All-trans* retinoic acid (*ATRA*) is the most relevant and functionally active metabolite of *Vitamin-A*. From a therapeutic standpoint, *ATRA* is the first example of pharmacological agent exerting its anti-tumor activity via a cell differentiating action. In the clinics, *ATRA* is used in the treatment of Acute Promyelocytic Leukemia, a rare form of myeloid leukemia with unprecedented therapeutic results. The extraordinary effectiveness of *ATRA* in the treatment of Acute Promyelocytic Leukemia patients has raised interest in evaluating the potential of this natural retinoid in the treatment of other types of neoplasias, with particular reference to solid tumors.

The present article provides an overview of the available pre-clinical and clinical studies focussing on *ATRA* as a therapeutic agent in the context of breast cancer from a holistic point of view. In detail, we focus on the direct effects of *ATRA* in breast cancer cells as well as the underlying molecular mechanisms of action. In addition, we summarize the available information on the action exerted by *ATRA* on the breast cancer micro-environment, an emerging determinant of the progression and invasive behaviour of solid tumors. In particular we discuss the recent evidences of *ATRA* activity on the immune system. Finally, we analyse and discuss the results obtained with the few *ATRA*-based clinical trials conducted in the context of breast cancer.

**Graphical Abstract:**

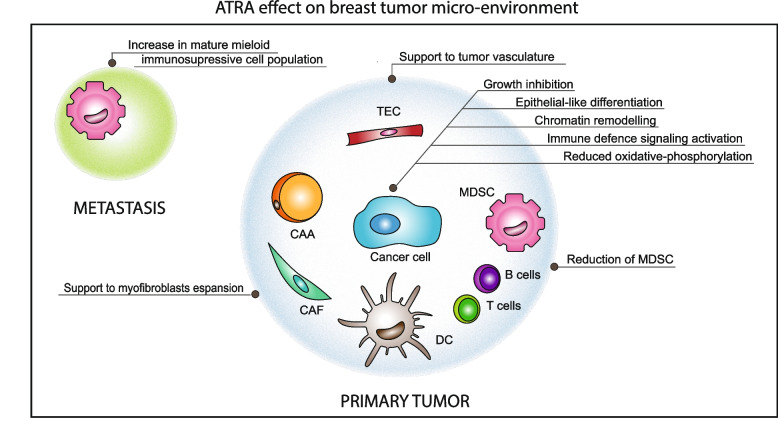

## Background

Vitamin-A consists of a group of organic compounds that includes retinol, retinal (also known as retinaldehyde), retinoic acid and several carotenoids [1, 2]. In humans, Vitamin-A is an essential nutrient and a fundamental component of standard diets. Vitamin-A is involved in numerous homeostatic functions, including the regulation of embryonic development as well as the maintenance of the immune system and vision in the adult [3, 4]. *All-trans* retinoic acid (*ATRA*) is considered to be the most relevant and functionally active metabolite of Vitamin-A [5]. In *APL* (*Acute Promyelocytic Leukemia*), *ATRA* is a standard component of the therapeutic regimen(s) which is used in the treatment of this rare type of myeloid leukemia. In this pathological context, therapeutic doses of *ATRA* restore the granulocytic differentiation program which is blocked in the leukemic blast, via an incompletely defined mechanism, which involves the degradation of the oncogenic fusion product deriving from a translocation involving chromosomes 15/17 and is known as *PML-RARα* [6]. As *RAR*α (*Retinoic Acid Receptor alpha*) is one of the nuclear receptors mediating the transcriptomic effects exerted by *ATRA*, the retinoid represents the first example of targeted anti-tumor agent showing therapeutic efficacy. The exceptional results obtained in *APL* has spurred interest in the evaluation of *ATRA* potential as a therapeutic agent in neoplastic diseases other than this myeloid leukemia, with particular reference to solid tumors such as breast cancer. In the present review article, we provide a brief summary of the pre-clinical and clinical data available in the literature on various issues relating to the anti-tumor activity of *ATRA* in mammary tumors. It is expected that the present review article may be of use in filling the gap between the available evidence on the therapeutic potential of *ATRA* in breast cancer as well as other solid tumors and the design of *ATRA*-based therapeutic strategies of clinical relevance.

## *ATRA* metabolism and signaling

The current section of the review provides information on the metabolism and signaling of *ATRA* that is of relevance to understand the molecular mechanisms underlying the anti-tumor action of the retinoid.

### ATRA up-take and metabolism in the normal and neoplastic cell

Even if nanomolar concentrations of *ATRA* have been detected in the bloodstream, the main source of intra-cellular *ATRA* is represented by serum retinol [7]. Circulating retinol is present in the bloodstream under the form of a complex with *RBP4* (*Retinol Binding Protein 4*) and *TTR* (*Transthyretin*). The concentration of retinol which can be determined in the circulating blood is in the low μM range [2]. Given the lipophilic nature of retinol and retinoid derivatives, the uptake of most of Vitamin-A metabolites occurs via diffusion through the plasma membrane. In some tissues, including breast cancer, the retinol/*RBP4* complex can be internalized into the cell via a plasma membrane protein known as *STRA6* (*Signaling Receptor And Transporter of Retinol STRA6*) [8].

**Fig. 1:**
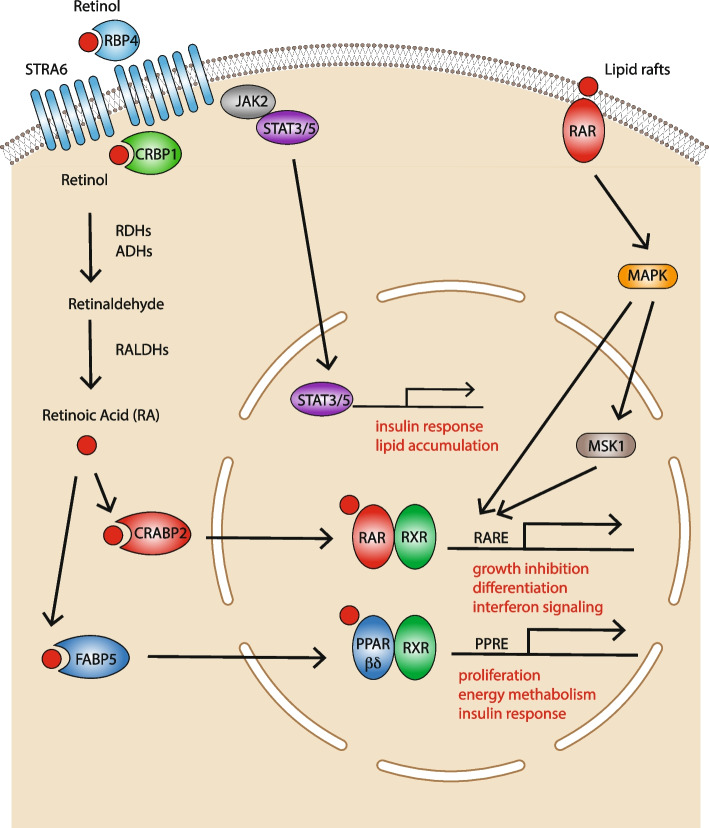
ATRA uptake inside the cell and intra-cellular mechanism of action. Dietary *Vitamin-A* is released into the blood as retinol which is complexed to *RBP4* (*Retinol Binding Protein 4*). The cellular uptake of retinol is mediated by *STRA6* (*Stimulated by Retinoic Acid 6*). Intracellular retinol is either stored as an ester or further oxidized into retinaldehyde by *ADH*s (*Alcohol Dehydrogenases*) or *RDH*s (*Retinol Dehydrogenases*). Retinaldehyde is further transformed into ATRA by *RALDH*s (*Retinaldehyde Dehydrogenases*). In the cytosol, ATRA can be transported to the nucleus by *CRABP2* (*Cellular Retinoic Acid Binding Protein 2*) or by *FABP5* (*Fatty Acid Binding Protein 5*). By converse, ATRA is targeted to degradation by *CRABP1* (*Cellular Retinoic Acid Binding Protein 2*). In the nucleus, ATRA binds to one of the indicated SNRs (Steroid Nuclear Receptors) generating an active transcriptional complex which interacts with the DNA regulatory regions of target genes containing *RARE*s (*Retinoic Acid Responsive Elements*) or *PPREs (Peroxisome Proliferator Responsive Elements*). In breast cancer cells, ATRA binding to the *SNR* heterodimers *RAR*/*RXR* (*Retinoic Acid Receptor*/*Retinoic X receptor*) triggers growth inhibitory/differentiating effects while ATRA binding to *PPAR*/*RXR* heterodimers (*Peroxisome Proliferator Activated Receptor*) sustains proliferation. ATRA exerts non-genomic effects via interactions with membrane bound *RAR*s. These interactions activate *MAP-kinase* cascades which concur to the activation of *RARE* dependent target genes. Retinol bound *RBP4* can also trigger intra-cellular signals by activating the *STAT3*/*STAT5* transcription factors

*RBP4*- and *TTR*-bound retinol are uptaken by the normal and the neoplastic cell (Fig. 1). Once inside the cell, retinol is converted into *ATRA* by a two step-reaction. In the first step, *RDH*s (*Retinol Dehydrogenase*s) or *ADH*s (*Alcohol Dehydrogenase*s) metabolize retinol into retinaldehyde and these enzymatic reactions are reversible. This is followed by the irreversible oxidation of retinaldehyde into *ATRA* which is carried out by *RALDH*s (*Retinaldehyde Dehydrogenase*s) [1]. Since all retinol derivatives are lipophilic, as mentioned above, these molecules are present in the cytosol under the form of complexes with specific binding proteins. Indeed, eukaryotic cells contain different intracellular proteins capable of binding *ATRA*, retinol and/or retinaldehyde. Newly synthetized *ATRA* interacts with *CRABPI* or *CRABPII* (*Cytosolic Retinoic Acid Binding Protein I* and *II*), two well-known cytosolic proteins. In general, *CRABPI* is considered to promote *ATRA* degradation, while *CRABPII* is believed to translocate *ATRA* into the nucleus where it binds *RAR*s and *RXR*s (*Retinoid X Receptor*s), which are ligand-dependent transcription factors belonging to the family of *SNR*s (*Steroid Nuclear Receptors*). *ATRA* degradation is carried out by a few *Cytochrome P-450 Hydrolases* (*CYP26A1*, *CYP26B1* and *CYP26C1*) and the process plays a major role in the control of the intracellular levels of the retinoid. The homeostatic concentrations of intracellular *ATRA* fall within the low nanomolar range [2]. In physiological conditions, the presence of such endogenous concentrations of *ATRA* is of fundamental importance for the maintenance of Vitamin-A dependent gene-expression and signaling. In terms of activity on target cells, *ATRA* can exert its physiological action in both an autocrine and a paracrine manner [9].

### ATRA intracellular signaling

*ATRA* intracellular signaling occurs via genomic and non-genomic pathways. In the classical genomic pathway, *ATRA* acts as a ligand for transcription factors complexes that activate/repress a plethora of target genes [5]. *ATRA* exerts also many non-genomic effects, which include the activation of various kinase signaling pathways [10] (Fig. 1).

In the classical genomic pathway, the six *SNR*s, *RAR*α, *RAR*β, *RAR*γ, *RXR*α, *RXR*β and *RXR*γ are believed to mediate the vast majority of *ATRA*-dependent transcriptional effects. The transcriptionally active forms of these *SNR*s consist of different *RAR*/*RXR* heterodimers in which *RAR* acts as the *ATRA* ligand-binding moiety. The ligand bound *RAR*/*RXR* heterodimers interact with *RARE* (*Retinoic Acid Responsive Element*) DNA sequences, which are generally located in the promoter region of retinoid target genes. *ATRA* binding to *RARs* generates a conformational change of the *RAR/RXR* complexes*.* This altered conformation modulates the association of the *RAR*/*RXR* heterodimers with the corresponding co-repressors/co-activators and it drives the epigenetic modifications which govern target gene transcription [10, 11]. The fundamental role of *RAR*s in *ATRA* signaling is supported by a recent study based on the use of the *CRISPR*/*CAS9* strategy and aimed at silencing *RAR*s in murine embryonic stem cells [12]. The results obtained demonstrate that *RAR*s are essential for the regulation of all the transcripts whose expression is controlled by *ATRA*. Besides *RAR*s, *ATRA* is known to bind and modulate the transcriptional activities of other members of the *NR* family, including *PPARβ-δ* (*Peroxisome Proliferator-Activator Receptor* β/δ [13], *ROR*β/*NR1F2* (*RAR-related Orphan Receptor Beta* or *Nuclear Receptor 1F2* [14], *COUP-TFII*/*NR2F2* (*Chicken Ovalbumin Upstream Promoter-Transcription Factor II* or *Nuclear Receptor 2F2* [15] and *TR4*/*NR2C2* (*Testicular Receptor 4* or *Nuclear Receptor 2C2* [16]. In breast cancer, *ATRA*-liganded *RAR*/*RXR* heterodimers are purported to mediate growth inhibition, whereas the *PPAR*β-δ/*RXR* heterodimer is believed to induce proliferation [13, 17]. *ATRA* is transported to the nucleus by the above mentioned *CRABP2* and the *FABP5* (*Fatty Acid Binding Protein 5*) cytosolic receptors. While *CRABP2* delivers *ATRA* to the *RAR*/*RXR* heterodimers, the retinoid is delivered to the *PPARβ-δ/RXR* heterodimer by *FABP5* [13]. The relative intracellular levels of *CRABP2* and *FABP5* determine the net effect of *ATRA* on cell proliferation.

The non-genomic signaling pathway of *ATRA* involves the activation of two complex intracellular processes. The first process involves the activation of specific protein kinases which lead to a cascade of post-transcriptional modifications of *RAR*s as well as the corresponding co-repressors, co-activators and chromatin remodeling targets [18]. This results in fine-tuning of the transcriptional effects exerted by *ATRA* on retinoid target genes. Interestingly, a proportion of the various *RAR* proteins associates with the membrane lipid rafts and it is supposed to activate different types of kinases depending upon the cellular context. For instance, in some epithelial cells, including breast cancer cells and tumor associated fibroblasts, *ATRA* binding of plasma membrane *RAR*α activates the *p38MAPK* (*p38 Membrane Associated Protein Kinase*). In this situation, *ATRA* bound *RAR*α has been shown to interact with the *G-protein* αq subunit, which supports *MAPK* activation resulting from the engagement of upstream signaling cascades, such as those triggered by *Rho GTPase*s [19]. In neuronal cells, *ATRA* stimulation of plasma membrane associated *RAR*(s) activates the *p42*/*p44 MAPK* [18]. In this cellular context, *ATRA* stimulated *MAPK*s trigger *MSK1* (*Mitogen and Stress-activated Protein Kinase-1*) phosphorylation and translocation into the nucleus, where the protein is recruited to the promoters of retinoid target genes and it modulates transcription. Another *ATRA*-dependent non-genomic process involves the activation of the *JNK2* (*Janus Kinase 2*)/*STAT3*-*STAT5* (*Signal Transducer and Activator of Transcription 3* and *5*) signaling pathway via the *STRA6* (*Signaling Receptor And Transporter of Retinol*) transporter. Indeed, *STRA6* is not only a retinol transporter, but it is also a signaling receptor, which can be activated by retinol-bound *RBP4*. In this context, *RBP4* mediated retinol transport induces the phosphorylation of *STRA6*, which results in the activation of the *JAK2*/*STAT3–5* signaling cascade causing the induction of *STAT* target genes [20]. *STRA6*-mediated retinol transport and cell signaling are inter-dependent and they both rely on intracellular retinol trafficking and metabolism. Hence, *STRA6* associates Vitamin-A homeostasis and metabolism to cell signaling, which results in the control of important biological functions such as insulin responsiveness [21].

## Direct effects of *ATRA* on breast cancer cells

The present chapter of the review aims at providing a brief summary of what is known in terms of the direct effects exerted by *ATRA* on the neoplastic cell, taking into consideration the results obtained in breast cancer.

### Anti-proliferative action of ATRA

Many studies describe the effects of *ATRA* on breast cancer cell lines and breast cancer mouse models [5]. In general, *ATRA* is reported to exert an inhibitory effect on the growth of the mammary tumor cell. In cell lines, *ATRA* triggers a growth arrest which is not associated with massive cytotoxic effects [22, 23]. Indeed, in the majority of the cell lines considered, apoptosis is only a late and secondary effect induced by *ATRA* [24]. Significantly, a considerable reduction in the growth of tumor cells is also observed in mice bearing xenografts of breast cancer cells [22, 23]. As to the *RAR*/*RXR* isoforms mediating the anti-proliferative action of *ATRA*, the data available are in line with a major involvement of *RAR*α. In fact, in culture of different breast cancer cell lines, *RARα* silencing impairs the anti-proliferative activity of *ATRA* [22, 25]. In addition, transgenic mice characterized by the *MMTV* (*Mouse Mammary Tumor Virus*) dependent expression of a dominant-negative *RAR*α mutant, which is characterized by a defective ligand binding domain (*RAR*α*G303E*), develop metastatic mammary adenocarcinomas [26]. Finally, the role played by *RARα* in mediating the anti-proliferative action of *ATRA* in breast cancer is supported by studies performed with *RARα* specific agonists. Indeed, in both cell cultures and mouse models of mammary tumors, *ATRA*-induced growth inhibition is recapitulated only by the *RAR*α agonist AM580, as *RAR*β and *RAR*γ agonists are generally ineffective in terms of anti-proliferative effects [22, 27–30].

In breast cancer, the anti-proliferative activity of *ATRA* is observed predominantly in tumors characterized by a luminal and *ER*^+^ (*Estrogen Receptor* positive) phenotype, while the basal and *HER2*^+^ (*Human Epidermal Growth Factor Receptor 2* positive) subtypes of this neoplastic disease are generally resistant to the retinoid [22, 31, 32]. The observation is supported by *ATRA*-sensitivity studies performed on *ER*^+^ breast cancer cell lines and it is consistent with the demonstrated crosstalk between *ER*α (*Estrogen Receptor alpha*) and *RAR*α. Indeed, *RAR*α is known to be a direct *ER*α target gene, which is induced upon estrogen treatment of breast cancer cells [33, 34]. In this cellular context, chip-seq studies indicate that the DNA binding of *RAR* and *ER*α throughout the genome is highly coincident and this coincidence is at the basis of a widespread crosstalk between *ATRA* and estrogen signaling, which results in an antagonistic regulation of breast cancer-associated genes [25, 35]. In its unliganded form, *RAR*α is part of the *ER*α transcriptional complex and it contributes to the proliferative activity of estrogens in *ER*^+^ breast cancer cells. Upon ligand binding, *RARα* acquires an opposite function and it acts as an inhibitor of estrogen target genes transcription. As already mentioned, basal and *HER2* enriched *ER*α^−^ negative cancer cell lines tend to be resistant to *ATRA*, although some exceptions to the rule are observed. For instance, *ATRA* exerts a significant anti-tumor action on *TNBC* (*Triple Negative Breast Cancer*) cells characterized by constitutive activation of the *NOTCH1* (*Neurogenic locus NOTCH Homolog protein 1*) cell membrane receptor. In this cellular context, *RAR*β-silencing studies provide evidence that the *RAR*α-dependent induction of *RAR*β contributes to *ATRA* sensitivity [27]. In addition, 23–32% of the human breast cancers characterized by amplification of the *ERBB2* gene (the locus encoding the *HER2* protein) show co-amplification of the *RAR*α gene (*RARA*). Interestingly, this subtype of breast cancer shows a remarkable sensitivity to *ATRA* and *RAR*α agonists, regardless of *ER*-positivity [23].

Among the genes known to play a relevant role in mammary tumors, *BRCA1* (*BReast CAncer gene 1*) and *PALB2* (*Partner And Localizer of BRCA2*), two breast cancer susceptibility genes whose mutations result in familial cases of the disease, seem to be required for RARα signaling [36]. *ATRA* recruits *BRCA1* and *PALB2* to the promoters of retinoid-responsive genes where the two proteins play an important role in the transcriptional responses to the retinoid. In the breast cancer cell line MCF-7*, ATRA* signaling requires *BRCA1* and *PALB2* for the modulation of all the retinoid regulated transcriptome including the classical retinoid-responsive *HOX*s (*Homeobox* genes) genes. In breast cancer cells, depletion of *BRCA1* or *PALB2* diminishes the anti-proliferative effect exerted by *ATRA*.

As to genes other than *BRCA1* and *PALB2* that may be involved in the anti-proliferative action exerted by *ATRA* in mammary tumors, important clues come from gene-expression and DNA-methylation studies. The gene expression studies that we performed in a large panel of breast cancer cell lines and we validated in tissue slices from breast cancer patients [37] led to the identification of numerous transcripts which are induced by *ATRA*. In addition, the data generated indicate that the anti-proliferative action exerted by the retinoid is proportional to the amount of induced transcriptome perturbations [37]. Transcriptomic analyses performed on these data indicate that *ATRA* activates signaling pathways related to: 1) cell-cycle control, such as chromatin organization and DNA repair; 2) antigen presentation; 3) interferon responses. The results obtained allowed us to develop a gene-expression signature which predicts the anti-proliferative response of breast cancer to *ATRA* [32]. Similar types of studies aimed at assessing the DNA methylation status of *TNBC* cells led to the identification of over 1400 sites, which are differentially methylated in *ATRA*-resistant relative to *ATRA*-sensitive cell lines. The methylation profile generated from these data is proposed as a predictor of ATRA anti-proliferative activity in *TNBC* [38].

### Cell-differentiating action of ATRA

*ATRA* is the first example of therapeutically useful differentiating agent and it stimulates the expression of differentiation markers in various types of cancer cells. With respect to this, *ATRA* has been shown to reverse the process of *EMT* (*Epithelial-to-Mesenchymal Transition*) in different cell lines originating from solid tumors. *ATRA* seems to reverse *EMT* by restoring the expression of *E-cadherin*, by downregulating the expression of *Vimentin* and by increasing cell-to-cell interactions [39–41]. In addition, *ATRA* has been reported to inhibit cancer cell invasion and metastasis in a variety of tumor types [42].

In breast cancer, the subpopulations of *LEP* (*Luminal EPithelial*) and *MEP* (*MyoEPhitelial*) mammary cells respond to *ATRA* in a different manner, as indicated by the results obtained in the model represented by the bi-cellular *LM38-LP* murine mammary adenocarcinoma cell line. Indeed, the *LEP* subpopulation responds with an increase in cell-cycle arrest and apoptosis. By converse, the *MEP* subpopulation responds with an induction of the senescence and adhesion processes, which results in a decrease of the cell invasive capacity [42]. In agreement with this last finding, a screening of organic molecules based on the immortalized breast mesenchymal cell line, *NAMEC*, led to the identification of *ATRA* and its synthetic analog, *TTNPB* (a potent and selective pan-*RAR* agonist), as pharmacological inducers of the *MET* (*Mesenchymal to Epithelial Transition*) process [43]. Indeed, the authors demonstrate that the two retinoids switch breast cancer cells from a mesenchymal state to an epithelial/luminal like state, causing a reduction of the metastatic potential. In this situation, no effect of *ATRA* or *TTNPB* on cell proliferation is observed.

In the in vitro model of tumor progression represented by the *MCF10F* cell line, *ATRA* re-differentiates early-stage transformed cells (restoring the branching phenotype in 3D cultures), while the retinoid is ineffective, when cells characterized by a more malignant state are considered. In this model, *ATRA* inhibits the expression of breast cancer-associated genes [44]. In immortalized mammary tumor cells, it is interesting to notice that *ATRA* exposure inhibits *EMT*, while *RAR*α overexpression exerts an opposite effect on the process. In the breast cancer model represented by *MCF10A* cells, *RAR*α overexpression disrupts the normal acinar structure of the 3D cultures, inducing features of the *EMT* status [45].

*ATRA* induces an epithelial differentiation pathway in the *HER2*^*+*^ *SKBR3* and *UACC812* cell lines, which are characterized by the co-amplification of the *ERBB2* and *RARA* genes. Tight and adherens junctions are formed or reorganized in *SKBR3* cells as a result. Moreover, in this cell line the retinoid inhibits migration triggered by the well-known *EMT* inducers *EGF* (*Epithelial Growth Factor*) and *Heregulin-1* [27]. Finally, it is interesting to notice that exposure of *SKBR3* cells to *ATRA* stimulates the expression of VE-cadherin and other endothelial genes. The observation suggests that *ATRA* can induce the trans-differentiation of cancer cells towards an endothelial-like phenotype which may be of significance for the homeostasis of the tumor vasculature [46, 47].

### ATRA and breast cancer cell metabolism

Cancer cells have to rely on metabolic adaptation to sustain proliferation and the consequent biomass production. Increasing evidence supports the idea that specific genetic and epigenetic alterations in cancer cells are required to maintain a pro-anabolic state of their metabolism [48].

On one hand, *ATRA* is expected to interfere with cancer cells metabolic pathways as a consequence of its ability to affect the proliferation and the differentiation state of *ATRA* sensitive cancer cells. On the other hand, *ATRA* itself is a metabolite of the retinol metabolic pathway.

In the *NB4* model of *APL* disease, *ATRA*-induced differentiation of the leukemic blasts causes metabolic reprogramming. Indeed, the retinoid activates aerobic glycolysis and it reduces OXPHOS-dependent *ATP* production [49]. Similarly in breast cancer cells, transcriptomic analysis identifies Oxidative Phosphorylation as a Hallmark gene-set enriched for genes down-regulated by *ATRA* in sensitive luminal and basal cell-lines [50]. This effect is associated with a decrease in the mitochondria pool generating deficits in the respiration/energy-balance of breast-cancer cells. In breast cancer, *ATRA* is also known to regulate the activity of well-known oncogenes and tumour suppressors affecting cell metabolism such as *AKT1* and *BCL-2* [23, 48–52].

Along with other well-known pathways such as amino acid metabolism, arachidonic acid metabolism, fatty acid metabolism and linoleic acid metabolism, retinol metabolism stands among the metabolic pathways differentially modulated in healthy individuals versus breast cancer carriers [53]. In addition, in vitro models indicate that alterations in cellular retinol metabolism contribute to differential retinoid responsiveness in normal human mammary epithelial cells versus breast cancer cells [54, 55].

## Effects of *ATRA* on the mammary tumor micro-environment

**Fig. 2:**
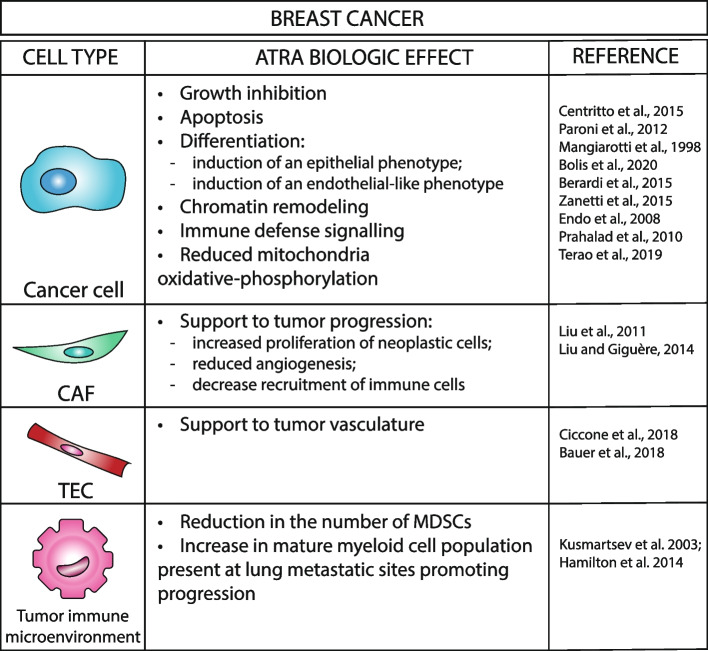
ATRA biological effects on the tumor micro-environment

The present chapter of the review focusses on the information available regarding the effects exerted by *ATRA* on the breast cancer micro-environment, which represents an important determinant of the progression and invasiveness of solid tumors (Fig. 2).

### Actions of ATRA on cancer associated fibroblasts

Fibroblasts play a key role in the production of the *ECM* (*ExtraCellular Matrix*), which is a major regulator of tissue repair and influences the function of epithelial, endothelial and immune cells [56, 57]. Normal fibroblasts are suggested to exert an anti-cancer action by preventing tumor expansion. In contrast, *CAF*s (*Cancer Associated Fibroblasts*) are considered to retain a tumor-suppressive function during the early stages of tumorigenesis, while they seem to increase the growth and the invasive/metastatic properties of late-stage cancer cells. *CAF*s act via remodeling of the *ECM* and the release of signaling molecules (growth factors, cytokines, chemokines and matrix re-organizing enzymes), which support cancer cell proliferation, angiogenesis and immune escape [56, 57].

In breast cancer, fibroblast-driven retinoid signaling seems to stimulate tumor progression, as indicated by studies conducted in mouse models of *ErbB2*-induced mammary tumors [58]. Indeed, experimental work based on tissue recombination technologies suggests that stromal *RAR*β promotes mammary gland tumorigenesis. In *Rarb*^−/−^ mice (*RAR*β-null mice), *ErbB2*-induced mammary tumors display a decrease in the proliferation of neoplastic cells, as well as a diminution of the angiogenetic process, the levels of collagen and the recruitment of inflammatory cells. In addition, the tumor tissues are characterized by a decrease in chemokine expression as well as an increase of the apoptotic responses observed in cancer cells. In *ErbB2*-induced mammary tumors, *RAR*β was found expressed in a subpopulation of myofibroblasts and its loss dampens their expansion and activity. Fibroblast production of *CXCL12*/*SDF-1* was shown to promote tumor cell proliferation by the activation of the *CXCR4*/*ErbB2* axis. It is interesting to notice that the mammary fat pads of *RAR*β-null mice hosting *ErbB2*-transformed epithelium show the presence of small in situ ductal carcinomas. The observation supports the notion that the loss of *RAR*β is of no significance for the tumor initiation process activated by *ErbB2*. By converse, the absence of *RAR*β reduces the capacity of the surrounding stroma to provide the necessary micro-environment for the growth and invasion of tumor cells. Noticeably, *RAR*β expression distinguishes the tumor associated stroma from the adjacent normal stroma in laser-dissected stromal cells obtained from a small cohort of breast cancer patients [58]. The data reported for *ErbB2*-induced mammary tumors were confirmed also in the case of *Wnt1*-induced mammary tumorigenesis [59]. In this case, the absence of *RARβ* changes the composition and the expression profile of the tumor stroma sustaining mesenchymal traits in the tumor cells.

The lack of cell-specific markers and the diverse origin of *CAF*s suggest that these fibroblasts can be heterogeneous in terms of function, which is highly dependent on the tumor context. Thus, it is not a surprise that the *ATRA*-dependent regulation of *CAF*s causes tumor suppression in neoplastic diseases other than breast cancer. Stellate cells are a peculiar type of hepatic and pancreatic fibroblasts, which are capable of storing lipid droplets as well as *ATRA* and retinoid derivatives. The balance between activation and quiescence of stellate cells is involved in the control of pancreatic tissue homeostasis. In vitro and in vivo preclinical studies performed with appropriate mouse models indicate that *ATRA* induces the quiescence of pancreatic stellate cells, which results in a slowing of tumor progression [60]. In these models, *ATRA* suppresses force-mediated extracellular matrix remodeling and inhibits local cancer cell invasion [61]. The above mentioned observations provided the rationale for a completed phase I and an ongoing phase II clinical trial aimed at demonstrating that *ATRA* is a stromal-targeting agent which can be used in combination with Gemcitabine and nab-Paclitaxel (albumin and paclitaxel containing nanoparticles) for the treatment of pancreatic ductal adenocarcinoma [62]. It should be noticed that a study performed in *SCC* (*Squamous Cell Carcinoma*) and aimed at determining the nuclear receptors, which are involved in the regulation of *CAF* functionality, resulted in the identification of *RAR*β, *PPAR*β-δ, *VDR* (*Vitamin D Receptor*), *GR* (*Glucocorticoid Receptor*) and *AR* (*Androgen Receptor*) as key players in the attenuation of the invasiveness, proliferation, drug resistance, energy metabolism and oxidative stress observed in the neoplastic cell. In this study, the treatment of mice carrying subcutaneous xenografts of *SCC* cells and *CAF*s with combinations of cisplatin, *RAR*β and *AR* antagonists inhibits chemoresistance in recurring tumors [63].

### Actions of ATRA on cancer associated adipocytes

Overweight and obesity are associated with an increased and decreased risk of breast cancer in post-menopausal and pre-menopausal women, respectively [64]. In addition, the two phenomena correlate with an increased risk of breast cancer–specific mortality in both post- and pre-menopausal women. Interestingly, adipocytes constitute the main cellular component of the breast tissue and neoplastic cells are embedded in the adipocyte-rich micro-environment of the mammary gland [65]. This is at the basis of the multiple functional interactions between adipocytes and breast cancer cells.

*CAA*s (*Cancer Associated Adipocytes*) diverge from their normal counterparts both in terms of phenotype and function. The interplay between *CAA*s and cancer cells confers oncogenic properties to the tumor micro-environment, favoring angiogenesis as well as the proliferation and local/distant dissemination of tumor cells. The mechanisms underlying these pro-oncogenic responses seem to involve adipokine regulation, extracellular matrix remodeling, immune cell modulation and metabolic reprogramming. Indeed, cancer cells establish a metabolic symbiosis with *CAA*s, absorbing the lactate, fatty acids and glutamine produced by neighboring adipocytes [66]. No published data related to the effects exerted by *ATRA* on *CAA*s are available. Nevertheless, it is reasonable to hypothesize that the retinoid may influence the behavior of *CAA*s, as adipocytes play an important role in *Vitamin-A* storage/metabolism and *ATRA* influences the homeostasis of adipose tissue [2]. With respect to this last point, it is established that retinol is mobilized from adipocytes when the circulating levels of Vitamin-A are insufficient. In addition, *ATRA* inhibits *CEBP*β (*CCAAT-Enhancer-Binding Protein* *beta*)/*PPAR*γ-mediated adipocyte differentiation of pre-adipocytes and mesenchymal progenitor cells in culture [67–69] and this inhibitory effect on adipogenesis is observed also in the living mouse [21, 70]. Furthermore, *ATRA* impairs adipocyte differentiation of human adipose-derived stem cells [71]. Indeed, single-cell gene-expression studies indicate that *ATRA* signaling modulates the activity of a particular subtype of mammalian *ASPC*s (*Adipose Stem and Precursor Cells*), which exert anti-adipogenic activity and are known as *Areg*s (*Adipogenesis regulator*s) [72]. Finally, it is interesting to notice that the adipose tissue of breast cancer patients contains higher concentrations of retinol than the corresponding counterpart isolated from women bearing benign mammary tumors [73].

### Actions of ATRA on tumor vascularization

*ATRA* plays a crucial role in the development of the mammalian vascular system. Indeed, *ATRA* controls endothelial cell proliferation and vascular remodeling during the process of tissue angiogenesis [74, 75]. In matrigel implant models, *ATRA* enhances the growth and function of mature micro-vessels supporting the therapeutic potential of the retinoid in pathological contexts characterized by defective angiogenesis [76]. In the skin, the *RAR* agonist, tazarotene, has been shown to promote the process of wound-healing via stimulation of tissue regeneration and neo-angiogenesis [77, 78].

In the tumor tissue, hypoxic cancer cells stimulate the generation of a dysfunctional vasculature which supports tumor growth and hampers immune responses [79]. *TEC*s (*Tumor-associated Endothelial Cells*) are highly heterogeneous and differ from normal endothelial cells in terms of phenotype and function [80, 81]. *TEC*s are endowed with genetic abnormalities and they are characterized by high levels of proliferative and transcriptional activities. In addition, *TEC*s are involved in the generation of an immune-suppressive tissue micro-environment, as they contribute to the deficiency in vascular permeability and the significant modifications of the immune-modulatory signals that are observed in tumor tissues [79]. In preclinical models of *SCC* and *APL*, *ATRA* and its derivatives inhibit tumor related angiogenesis both directly, via actions on the cancer cell, and indirectly, via modulation of the endothelial cell function [82–85]. With respect to the direct effects of *ATRA* on endothelial cells, it is interesting to notice that the retinoid inhibits the endothelial-to-osteoblast transition, a process known to be involved in the process of prostate cancer metastatization to the bone [86]. In the context of breast cancer, there are no comprehensive studies on the effects exerted by *ATRA* on *TEC*s. Nevertheless, the role played by *ATRA* in the homeostasis of normal vessels suggests that the retinoid is likely to modulate the function of *TEC*s in the tissue. Indeed, *ATRA* controls the expression of genes, such as *VEGF* (*Vascular Endothelial Growth Factor*), *FGF* (*Fibroblast Growth Factor*), *TGF*-β (*Transforming Growth Factor-beta*) and *Hedgehog*, which are known to be involved in vascular patterning and morphogenesis [87]. Indeed, *ATRA* induces the expression of *HIF-1*α (*Hypoxia-Inducible Factor 1-alpha*) and *VEGF* in certain solid tumors and the *ATRA*/*HIF-1*α/*VEGF* pathway has been reported to promote tumor angiogenesis in *HUVEC* co-cultures and xenografts of the *MCF-7* breast cancer cell line [88]. Interestingly in the context of the *4T1* syngeneic breast tumor model, *ATRA* was shown to increase the number of pericytes associated with intratumor endothelial cells and to normalize the tumor vessel morphology [89].

### Actions of ATRA on the immune micro-environment

*ATRA* is involved in the regulation of the immune system and it plays a key role in the control of inflammatory responses [4]. With respect to this, Vitamin-A insufficiency, which affects 250.000–500.000 children worldwide, is associated with a significant rate of morbidity and mortality during common childhood infections [90]. Although increased susceptibility to childhood infections is believed to be partially due to an impairment of the protection afforded by the epithelial barrier to mucosal pathogens, dysregulation of the immune cell responses is also likely to play a significant role [91]. Indeed, different studies suggest that Vitamin-A metabolites support the differentiation and functional activity of immune cells including dendritic cells/macrophages and lymphocytes [4] [92]. In terms of induced immune-responses, *ATRA* has been described to exert both tolerogenic and pro-inflammatory effects [4, 92]. With respect to this, the main evidence comes from studies performed in the gut of mice exposed to commensal microorganisms and food antigens. In this setting, *DC*s (*Dendritic Cells*) play a pivotal role in antigen processing and in transferring micro-environmental cues to T/B-lymphocytes. Noticeably, these two processes are of fundamental importance for both the activation of immune responses and for the maintenance of immune tolerance [93]. *ATRA* has been shown to control the genetic program which regulates the maturation of the gut-homing precursors of *DC*s [94, 95]. In the intestine, *DC*s and macrophages synthesize *ATRA*, which, in turn, stimulates the expression of gut-homing molecules in T- and B-lymphocytes as well as in *ILC*s (*Innate Lymphoid Cells*) [96–100].

In the context of immune regulation, it must be emphasized that *ATRA* has also been shown to control the effector functions of T-lymphocytes. Indeed, the local production of *ATRA* by *DC*s and macrophages promotes the conversion of naïve *CD4*^*+*^ T-cells into *iTreg*s (*Induced regulatory T-cells*) [101–106]. In addition, *ATRA* inhibits the *IL*-6 driven induction of pro-inflammatory *Th17* cells [104–107]. Finally, the exposure of intestinal mucosa B-lymphocytes to *ATRA* promotes *IgA* class switching, a process which is required for optimal protection against intestinal pathogens [99, 108].

Although *ATRA* is predominantly known for its tolerogenic function, the retinoid can also trigger pro-inflammatory effects. Indeed, *ATRA* has been shown to evoke a tolerogenic or a pro-inflammatory effect depending on the micro-environment and the synergizing cytokines immune cells are exposed to. In physiological conditions, *ATRA* seems to represent a key determinant of the *Treg*-*Th17* balance, favoring the differentiation of naïve T-lymphocytes into *Treg*s. At physiological concentrations, *ATRA* is also a critical determinant in the development of *Th17* cells [109]. Upon infection, the local increase in the levels of *IL-1* counteracts the *ATRA*-dependent inhibition of *Th17* cells, while the cytokine enhances *IL-6* responses, tilting the *Treg*-*Th17* balance towards the *Th17* arm [110]. In addition, *ATRA* is required to elicit the pro-inflammatory responses of *CD4*^*+*^ T-cells to infection and mucosal vaccination [91]. In models of vaccination and allogeneic graft rejection, whole body imaging demonstrates that *ATRA* signaling is temporally and spatially restricted to the site of inflammation. Conditional ablation of *ATRA* signaling in T-lymphocytes arrests inflammation by altering the function, migration, and polarity of these cells [109]. In an intestinal environment undergoing an *IL15-*dependent stress, *ATRA* acts as an adjuvant in promoting rather than inhibiting the cellular and humoral inflammatory responses to food antigen. In fact, in these conditions, *ATRA* decreases and stimulates the polarization of *Treg* and *Th1* cells, respectively [111]. Finally, in the context of vaccinia virus infection, *ATRA* is required for the optimal differentiation of effector and effector memory *CD8*^*+*^ T-cells [112].

The relevance of *ATRA* for the cross-talk between tumor and immune cells is supported by an elegant study performed with *B16* melanoma tumor models. Indeed, the study provides evidence on the specific activation of *ATRA* driven transcription at the site of tumor growth. In particular, the work identifies *ATRA* signaling as a critical step for the clonal proliferation of *CD8*^*+*^ T-cells and for the inhibition of tumor growth [113]. A further investigation performed in a mouse model of colitis-associated colon cancer demonstrates a marked deficiency in the levels of *ATRA* observed in the colon due to alterations in the metabolism of the retinoid which are mediated by microbiota-induced intestinal inflammation [114]. In this context, *ATRA* seems to exert an anti-tumor action via modulation of the immune micro-environment. In fact, inhibition of the *ATRA*-dependent signaling pathway promotes tumorigenesis, whereas *ATRA* supplementation reduces the tumor burden. The beneficial effect exerted by *ATRA* on the tumor burden is mediated by cytotoxic *CD8*^*+*^ T-cells, which are activated by *MHCI* up-regulation in colon cancer cells. In contrast, a paper focusing on the tumor micro-environment of sarcomas supports the idea that *ATRA* exerts an oncogenic action. In fact, the levels of *ATRA* are reported to be higher in mouse sarcoma as compared to the normal mesenchymal tissues [115]. In addition, tumor cells evade immune responses by producing *ATRA*, which stimulates and inhibits the differentiation of tumor-associated monocytes into immunosuppressive macrophages and *DC*s, respectively. As a consequence, intra-tumor injection of a pan-*RAR* antagonist, which suppresses *ATRA* activity in the tumor micro-environment, promotes monocyte differentiation into antigen presenting cells, enhancing anti-tumor T cell responses.

It is believed that cancer cell survival and proliferation are sustained by stromal cells of the neoplastic micro-environment whose function has been hijacked to allow tumor progression [116]. In the case of immune cells, tumor-secreted factors prevent normal myeloid cell differentiation, which leads to the accumulation of a heterogeneous population of immune-suppressive immature myeloid cells known as *MDSC*s (*Myeloid Derived Suppressor Cells*). *MDSC*s are recruited to the primary tumor and metastatic micro-environment from the bone marrow and secondary lymphoid tissues (e.g. spleen, lymph nodes) via the blood stream [117]. Evidence obtained in both tumor bearing mice and cancer patients supports the idea that the presence of *MDSC*s sustains the survival of primary and metastatic cancer cells. *MDSC*s are capable of inhibiting the immune responses mediated by T- and B-lymphocytes as well as *NK* (*Natural Killer*) cells. Thus, inhibition of the immunosuppressive properties of *MDSC*s can improve anti-tumor responses [117]. Many reports support a role for *ATRA* in the modulation of *MDSC* (Table 1). Early studies performed in appropriate mouse models indicate that *ATRA* promotes the differentiation of *MDSC*s into *DC*s and macrophages. The phenomenon seems to be at the basis of *ATRA* anti-tumor action [118, 119]. With respect to this last contention, it should be emphasized that the above-mentioned mouse models are characterized by the fact that *ATRA* is capable of increasing only the anti-tumor action of vaccines, as the retinoid exerts no direct effect on tumor cell proliferation. Indeed, the sole administration of combinations between *ATRA* and vaccines exerts an anti-tumor effect, which results in the inhibition of tumor growth. The reduction in the number and activity of *MDSC*s induced by the combination of *ATRA* and vaccines has also been observed during the course of a clinical trial performed in patients affected by extensive-stage small-cell lung cancer [120]. The data mentioned above are further supported by a study conducted with a mouse *MC38* colon carcinoma model [121]. In this model, combinations of *ATRA* and radiotherapy induce the differentiation of tumor infiltrated monocytes into inflammatory *iNOS/TNFα-*producing macrophages, contemporaneously enhancing the priming/activation of tumor associated *CD8*^*+*^*/CD4*^*+*^ T-cells. The work provides evidence for a positive feedback loop between inflammatory macrophages and T-cells, which results in an inhibition of tumor growth at both the local and systemic level. Indeed, the combination of *ATRA* and ionizing radiation abrogates tumor re-growth after re-implantation of cancer cells into cured animals and it delays the growth of distant and non-irradiated co-implanted tumors. *ATRA* and immune-checkpoint inhibitors represent an additional example of retinoid-based combination therapy involving modulation of the immune micro-environment [122–124]. In mouse models of mesothelioma and fibrosarcoma, *ATRA* reduces the number of peripheral *MDSC*s and it induces an interferon-driven inflammatory tumor micro-environment enriched in *CD8*^*+*^ T-cells. This results in the sensitization of tumor cells to immune-checkpoint inhibitors [122]. Consistent with this, in a model of non-small-cell lung cancer resistant to immune-checkpoint inhibitors, *ATRA* restores sensitivity to anti-*PD1* agents stimulating tumor-specific T-cell dependent immunity [123]. The *MDSC* suppressive and *CD8*^*+*^ T-cell activating properties of *ATRA* are supported by recent phase II clinical trials combining the retinoid with ipilimumab or pembrolizumab in melanoma [125, 126]. The results obtained in sarcoma tumor models are in apparent contrast with the stimulatory effects exerted by *ATRA* on the anti-tumor action of checkpoint inhibitors. In this model, intra-tumor injection of a pan-*RAR* antagonist enhances the anti-tumor activity of checkpoint inhibitors via promotion of an immune stimulatory tumor micro-environment [115].

**Table 1:** Preclinical and clinical studies supporting ATRA antitumor activity via immunomodulation of the tumor micro-environment

STUDY	CO-TREATMENT	POSOLOGY	CANCER TYPE/MODEL	RESULTS	ATRA ACTIVITY	REF
Preclinical	Vaccination	1) For immunization of the MethA fibrosarcoma tumor model, dendritic cells (infected with adenovirus encoding full-length wild-type p53) were injected s.c. into mice (4x10^5^ cells/mouse) on days 5, 10, and 15 after tumor cell inoculation. ATRA pellet (21-day release, 5 mg) was implanted on day 7 after tumor cell injection. 2) In the DA3-HA mammary tumor model, ATRA (21-day release, 5 mg) was administered 4 days after tumor inoculation. HA based vaccinia (1x10^7^ PFU) was injected 15 days after tumor implantation. 3) C3 fibrosarcoma tumor-bearing mice were treated with three rounds of immunization with control or specific peptide (50mg) at day 5, 7 and 17 after tumor inoculation. At day 7, ATRA (21-day release, 5 mg) or placebo pellets were implanted.	Breast cancer, fibrosarcoma-syngeneic mouse models	ATRA increases the antitumor effect of vaccination	1) ATRA decrease the number of MDSCs and induces their differentiation; 2) ATRA reduces CD4^+^ induced tumor tolerance; 3) ATRA increase CD8^+^T cell response	Kusmartsev et al., Cancer Res, 2003 [118]
Preclinical	Vaccination	MC38-CEA colon-adenocarcinoma or B16-OVA melanoma tumors were implanted, and animals were vaccinated against CEA or OVA (200 mg)combined with 20 mg of CpG-Oligonucleotid 1668 and 0,2 mg of anti-Galactosylceramide (aGC) after 10 days. ATRA treatment was administered simultaneously or with a 3 days delay (500 ng /day /mouse). Treatment lenght was 8 and 13 days for MC38-CEA or B16-OVA models respectivelly.	Colon adenocarcinoma; melanoma-syngeneic mouse models	ATRA potentiates tumor vaccination if administered later after first vaccination	1) ATRA treatment inhibits the suppressive capacity of monocytic MDSCs; 2) ATRA induces elevated frequencies of antigen-specific and functional CD8^+^ cytotoxic T lymphocytes	Heine et al., Oncoimmunology, 2017 [127]
Preclinical	Radiation and PD-L1 blockade	MC38 colon-adenocarcinoma, B16/F1 melanoma and Renca renal derived tumors were irradiated (15Gy) and ATRA was administered by oral gavage for 10 days. In the MC38 model, anti–PD-L1 antibody was administered intraperitoneally on days 0, 3, and 7 after radiation at 200 μg per mouse.	Colon adenocarcinoma, melanoma, renal carcinoma-syngeneic mouse models	ATRA potentiates the antitumor effect of ablative radiation (IR)	1) IR and ATRA treatment lead to an abscopal response enhanced by PD-L1 blockade; 2) IR and ATRA induces inflammatory macrofages; 3) IR and ATRA increase T cell response	Rao et al., Sci Immunol, 2021 [121]
Preclinical	CTLA4 and PD-L1 blokade	AB1-HA mesotelioma bearing mice were treated with aCTLA-4 on day 7 and aPD-L1 on day 7, 9 and 11 post tumor cell inoculation. ATRA treatment was administered simultaneously or 3 days prior the ICI treatment at 10 mg/kg for 9 consecutive days. AB1-HA bearing mice were treated with anti-GITR or anti-CD40 (anti-OX-40) antibodies (day 7 post tumor cell inoculation), with or without ATRA (10 mg/kg) beginning 4 days prior ICI administration to day 13 post tumor inoculation. Mice bearing WEHI164 fibrosarcoma were treated with anti-CTLA4 or anti-PD-L1 on day 11 post tumor injection with or without ATRA (10 mg/kg, from day 8 to day 16 post tumor injection).	Mesotelioma, fibrosarcoma-syngeneic mouse models	ATRA improves the anti-tumour response to anti CTL4, anti PDL1, anti-GITR and anti-OX40. ATRA /ICI combination efficacy is schedule-dependent; ATRA was most effective at increasing the αCTLA-4/αPD-L1 mediated antitumour response when given 3 days prior to ICI	1) ATRA induces an inflammatory, Interferon-driven tumour microenvironment; 2) ATRA further enhances the inflammatory tumour microenvironment induced by ICI; 3) activation of inflammation distinguishes responders from non-responders following ATRA/ICI combination therapy	Tilsed et al., Front Oncol, 2022 [122]
Preclinical	PD-1 blockade	ATRA (200 μg/mouse) was administered daily, starting on day 5 post tumor inoculation (KPL-3M cells). Anti-PD-1 antibody (200 μg) was dministered 2–3 times per week for 5 doses starting from day 7 post inoculation.	LKB1-Deficient Non–Small Cell Lung Cancer- genetically engineered syngeneic murine models	ATRA potentiates the antitumor efficacy of anti PD1	1) ATRA suppresses the proliferation and function of G-MDSCs; 2) ATRA enhanced T-cell infiltration, proliferation, and activation; 3) combination therapy with ATRA and anti–PD-1 induces systemic tumor-specific immune memory	Li et al., Cancer Res, 2021 [123]
Preclinical	PD-L1 blokade	Tumor bearing mice were treated with ATRA (7.5 mg/kg) daily from day 6 post U14 cells injection. Anti-PD-L1 antibody (10 mg/kg) was administered on day 6, 9 and 12 and mice were sacrificed on day 21 post tumor cells inoculation.	Cervical cancer-syngeneic mouse models	ATRA enhances the antitumor efficacy of anti PDL1	1) ATRA partially reverses MDSCs suppression; 2) ATRA enhances CD8^+^ T cell infiltration	Liang et al., Scientific Reports, 2022 [128]
Clinical trial	Vaccination	Patients were randomized into three arms: Arm A (standard of care control patients or observation), Arm B (patients treated with p53 vaccine only) and Arm C (patients treated with vaccine in combination with ATRA, 150 mg/mq for 3 days beginning one day prior to each vaccine). Each vaccine consisted of 2–5 × 10^6^ autologous dendritic cells expressing p53 after infection with adenovirus encoding full-length wild-type p53 gene. Cells were injected intradermally, at two-week intervals three times (three vaccine doses). Patients were restaged approximately 2 weeks after the 3rd vaccine dose. Patients without disease progression underwent a second leukapheresis and were then vaccinated 3 more times at 4-week intervals.	Small cell lung cancer, extensive stage	ATRA improves the immune response to vaccination	1) ATRA decreases MDSCs; 2) ATRA increases Granzyme B positive CD8^+^T cells	Iclozan et al., Cancer Immunol Immunother, 2013 [120]
Clinical trial	CTLA4 blokade	Ipilimumab (anti CTL4) was administered in four adjuvant infusions of 10 mg/kg for stage III patients or four infusions of 3 mg/kg for stage IV patients every three weeks in the Ipilimumab arm. Patients in the Ipilimumab plus ATRA arm were treated with ATRA at 150 mg/mq at days −1, 0, and +1 from Ipilimumab infusion.	Melanoma, stage III and IV	The addition of ATRA to standard of care Ipilimumab therapy appears safe	1) ATRA significantly decreased the frequency of circulating MDSCs	Tobin et al., Int Immunopharmacol, 2018 [125]
Clinical trial	PD-1 blockade	Pembrolizumab (anti PD-1) was administered at a fixed dose regimen of 200 mg every 3 weeks for 5/6 cycles. ATRA (VESANOID) at 150 mg/mq was used for three days surrounding each (days −1, 0, and +1) of the first four infusions of pembrolizumab.	Melanoma, stage IV	The combination was well tollerated. The overall response rate was 71%, with 50% of patients experiencing a complete response, and the 1-year overall survival was 80%	1) ATRA significantly decreased the frequency of circulating MDSCs	Tobin et al., Clin Cancer Res, 2023 [126]

In breast cancer, the treatment of mammary DA3 tumor-bearing mice with *ATRA* causes a significant reduction in the number of *MDSC*s [118]. However, administration of *ATRA* as a single agent does not exert any effect on the growth of the primary tumor instead improved immune response to vaccination. In particular, the retinoid reduces the expansion of tumor-associated immature myeloid cells by triggering their differentiation into mature *DC*s, macrophages, and granulocytes. Moreover, in an experimental model of mammary tumor vaccination, *ATRA* activity reverts tumor-induced *CD4*^*+*^ T-cell tolerance [118]. The ability of *ATRA* to improve antitumor immune responses and to enhance the effect of vaccination was described also in other tumor types [118]. *ATRA* has also been reported to modulate immune cell composition in the metastatic niche. Indeed, the retinoid enhances the metastatic behavior of breast tumors obtained by transplantation of the mouse *4T1* and *4TO7* cell lines in immune competent mice. This effect has been ascribed to a change in the composition of the myeloid cell population present in the specific metastatic sites. In these models too, *ATRA* retains the ability to promote the differentiation of myeloid cells. However, in the lung metastatic sites, *ATRA* drives the differentiation of immature myeloid cells into a macrophage lineage with stronger immunosuppressive ability [129]. In other tumor contexts, such as osteosarcoma, *ATRA* prevents the metastatic spread of cancer cells by inhibiting the activity of inflammatory macrophages [130]. In animals transplanted with the mouse *4T1* and *TS/A* mammary adenocarcinoma cell lines, *ATRA* administration enhances the effects of antiangiogenic agents [89]. In this experimental setting *ATRA* reduces the *MDSC* increase induced by a monoclonal antibody targeting murine *VEGFR2*. Moreover, the retinoid contributes to the stabilization of the tumor endothelium exerting vessel-normalizing effects, which supports the antitumor effect of the anti-angiogenic compound. Interestingly, *ATRA* treatment rescues the hypoxic conditions induced by this antibody.

Altogether the above data support the notion that *ATRA* potentiates or impairs cancer progression by acting on the immune system depending upon the costimulatory conditions used.

## Activity of *ATRA* in association with current breast cancer treatments

Various pre-clinical studies indicate that *ATRA* improves the efficacy of current breast cancer treatments. For instance, *ATRA* has been used in association with the gold standard therapies for *ER*^+^ and *HER2*^+^ breast cancer. As far as *ER*^*+*^ tumors, *ATRA* potentiates the antiproliferative activity of tamoxifen, a well-known selective ER modulator used as a therapeutic agent in the clinics [51]. As the unliganded form of *RARα* is part of the *ERα* transcriptional complex, the cross-talk between *RARα* and *ERα* is thought to be at the basis of the observed antitumor efficacy of *ATRA*. Upon *ATRA* binding, *RARα* inhibits the proliferative effect trigger by the estrogen [25]. As far as *HER2*^*+*^ tumors, *ATRA* synergizes with the anti-*HER2* therapeutic agents trastuzumab and lapatinib, two clinically useful *HER2* kinase inhibitors [23, 131]. In particular, *ATRA* potentiates the *HER2* phosphorylation inhibition induced by lapatinib [23].

Immunotherapy (Atezolizumab and Pembrolizumab) are approved for the treatment of *TNBC* and metastatic *HER2*^*+*^ tumors [132]. Specific studies in breast cancer are not available, although *ATRA* regulates tumor associated immune cells in various types of solid tumors (Table 1). This is the basis of the benefits observed with combinations of *ATRA* and immunotherapy, which supports the idea that *ATRA* + immunotherapeutics may be effective in breast cancer as well.

*CDK4/6* inhibitors were shown to improve the survival in patients with metastatic breast cancer [132]. As a consequence, combinations of *CDK4/6* inhibitors and endocrine therapeutic agents represent the new standard of care for first and second line treatment of advanced *ER*^*+*^*/HER2*^*−*^ breast cancer. *ATRA* co-operates with the *CDK4/6* inhibitor, Palbociclib, at the transcriptional level, resulting in additive anti-proliferative and differentiating effects in neuroblastoma cells [133]. Studies on breast cancer models are necessary to support a possible role of *ATRA* also in this type of tumor.

Adjuvant radiotherapy is an essential component of breast cancer treatment. Although clinical trials aimed at assessing the benefit of *ATRA* administration in this setting are not available in the scientific literature, there is preclinical evidence indicating that the retinoid enhances radiation efficacy. Indeed, in mouse cancer models, combinations of *ATRA* and radiation therapy (ionizing radiation, 15Gy) reprogram intra-tumor myeloid cells, resulting in an enhancement of systemic anti-tumor immunity and a stimulation of immune checkpoint inhibitor (*ICI)* therapeutic activity [121] (Table 1).

In addition, several studies provide evidence of synergistic interactions between *ATRA* and chemotherapeutic agents [134]. For instance, pre-clinical studies support the idea that *ATRA* sensitizes breast cancer cells to paclitaxel and adriamycin, two chemo-therapeutic agents commonly used in the treatment of this tumor [135].

In recent years, several specific pre-clinical studies aimed at developing nanocarriers permitting the contemporaneous delivery of *ATRA* and chemotherapeutic agents have been conducted. In the *4T1* mouse metastatic breast cancer model, micellar nanoparticles containing combinations of *ATRA* and doxorubicin were administered in a post-operatory setting to reduce inflammation and *MDSC* recruitment [136]. These nanoparticles suppress post-operative recurrences and pulmonary metastases. In a orthotopic xenograft model of *TNBC*, *ATRA* and doxorubicin containing nanoparticles stimulate the differentiation of cancer stem cells [137]. This reduces the tumor initiating activity of cancer stem cells and increases their sensitivity to doxorubicin. In the same model, doxorubicin-mediated cytotoxicity is potentiated by *ATRA* driven differentiation and Entinostat (histone-deacetylase-inhibitor) dependent epigenetic remodeling [138]. Similarly, *ATRA* sensitizes breast cancer cells to paclitaxel [135]. Co-delivery of *ATRA* and paclitaxel using albumin-bound paclitaxel nanoparticles generates a significantly improved anti-metastatic effect [139]. Notably, co-delivery nanoparticles exhibit more pronounced therapeutic effects than the combination of the two free drugs. Another proposed nanodrug formulation consists of *ATRA* and irinotecan along with the photothermal agent *IR825* [140]. Indeed, the combination of *ATRA* with a chemotherapeutic drug (irinotecan) induces the differentiation and death of breast cancer stem cells, while *NIR* (Near to InfraRed) laser irradiation triggers a photothermal effect. In the mouse *4T1* breast cancer model, this combination causes a potent antitumor effect, which is superior to the effect observed with each single agent or double combination.

In conclusion, the results summarized above support the idea that *ATRA*-based therapeutic combinations with other anti-tumor agents may be of clinical significance in the management of breast cancer.

## *ATRA* in breast cancer clinical trials

The current chapter aims at providing a brief summary of the data supporting the clinical potential of *ATRA* in the management of breast cancer. Indeed, the chapter focusses on the results obtained with clinical trials involving the use of this retinoid.

The available experimental evidence supports the idea that *ATRA* exerts a general and direct onco-suppressive action in breast cancer cells. By converse, the effects exerted by *ATRA* on the tumor micro-environment are dichotomic, as the retinoid causes both oncogenic and onco-suppressive responses. Despite the large number of pre-clinical studies performed with *ATRA* there are only four registered clinical trials (*ClinTR1–4*) on the use of the retinoid as a therapeutic agent in the oncological setting (Table 2). *ClinTR1* is based on the use of *ATRA* as a single therapeutic agent in the treatment of patients affected by hormone-refractory, metastatic breast cancer and the results obtained lead to the conclusion that the retinoid is devoid of significant anti-tumor activity. However, the trial suffers from a number of limitations, including the low number of patients (14 patients altogether) and the high degree of inter-patient variability in terms of *ATRA* blood levels, following drug administration [141]. Two other trials (*ClinTR2* and *ClinTR3*) provide data on the anti-tumor action exerted by combinations of *ATRA* and tamoxifen or chemotherapy in breast cancer. *ClinTR2*, which was performed in the context of *ER*^+^ mammary tumors [142], indicates that the maximal daily dose of *ATRA* that can be administered with tamoxifen is 190 mg/mq. In addition, *ATRA* must be administered using two weekly cycles per month. The results obtained indicate objective responses in a number of patients undergoing progression following treatment with tamoxifen alone. However, the study design and the limited number of patients enrolled (*n* = 25) does not permit to draw any significant conclusion on the anti-tumor activity of the retinoid. Similar to what was observed in the case of the clinical trial conducted by Sutton et al., a high degree of intra- and inter-patient variability in the plasma levels of *ATRA* are reported. *ClinTR3* is a small pilot study involving 17 patients suffering from late stage breast cancer (15/17 tumors = stage III/IV) who were exposed to different types of therapeutic protocols [143]. The study demonstrates that the combination of *ATRA* (45 mg/mq/day) and paclitaxel is well-tolerated. In addition, the combination of *ATRA* and paclitaxel results in a modest response rate. Indeed, the time-to-progression and survival rates observed in *ATRA* plus paclitaxel treated patients are similar to those reported for paclitaxel alone. Nevertheless, treatment with the combination of the two drugs is associated with a relatively high rate of stable disease (58.8%). *ClinTR4* is an ongoing trial (NCT04113863) aimed at evaluating the activity of *ATRA* in combination with anastrozole in postmenopausal women with newly diagnosed ER^+^/HER2^−^breast cancer.

**Table 2:** *ATRA* in breast cancer clinical trials

TRIAL	TREATMENT	PHASE	PATIENTS (n°)	DOSE	CANCER TYPE	ENDPOINT	RESULTS	REFERENCE
ClinTR1	ATRA	Phase II	14	ATRA administered orally at a dose of 50 mg/mq three times a day for 14 consecutive days of a 21-day cycle	Metastatic breast cancer	1) Evaluation of tumor growth; 2) characterization of ATRA pharmacokinetics	1) no significant antitumor activity; 2) high degree of interpatient variability in plasma levels of ATRA	Sutton et al., Cancer Chemother Pharmacol,1997 [141]
ClinTR2	ATRA plus tamoxifen	Phase I/II	25	ATRA administered orally at a dose of 70, 110, 150, 190, or 230 mg/mq/day on odd-numbered weeks (consecutive cohorts of 3–6 patients). Tamoxifen administered at 20 mg daily	Hormone-responsive advanced breast cancer	1) Determination of the maximum tolerated dose; 2) evaluation of tumor progression; 3) characterization of ATRA pharmacokinetics	1) doses up to 190 mg/mq were tolerable; 2) objective responses were observed, some in patients who had previously progressed while receiving tamoxifen; 3) high degree of intra and interpatient variability in plasma ATRA concentration	Budd et al., Clinical Cancer Res, 1998 [142]
ClinTR3	ATRA plus paclitaxel	Phase II	17	ATRA administered orally at 45 mg/mq PO daily for 4 days starting 2 days before a 1 h treatment with paclitaxel (Taxol, Bristol-Myers Squibb, Plainsboro, NJ) 80 mg/mq IV administered weekly for 3 weeks, repeated in 28 day cycles until disease progression or until no longer tolerated	Recurrent or metastatic breast cancer	1) Evaluation of toxicity, response; 2) determination of time to progression and survival	1) ATRA is a well-tolerated regimen; 2) Time to progression and survival rates similar to those reported for paclitaxel alone	Bryan et al., Invest New Drugs, 2011 [143]
ClinTR4	ATRA plus anastrozole	Phase II	n.d.	ATRA administered orally at 45 mg/mq/day for 28 days. Anastrozole administered orally at 1 mg daily	Operable Estrogen Receptor positive/HER2-negative early breast cancer	Evaluation of ATRA biological effect on: 1) percentage of Ki67; 2) tumor size reduction; 3) response rate.	n.d.	n.d.

Besides the clinical trials described above, it is worthwhile mentioning the publication of a small pre-operative study in locally advanced breast cancer. The main objective of this last study was to define the biological effects of *ATRA* following a 3-week administration in the presence and absence of tamoxifen or *IFN2*α (*Interferon 2*α) prior to the surgical remotion of the tumor [144]. Overall, *ATRA* administration exerts an influence on the tumor grade but it is devoid of effects on the cell cycle kinetics (G0-G1 phase) and cell proliferation (Ki67 levels). The lowest dose of *ATRA* producing a biological response is reported to amount to 15 mg/mq/day. *ATRA* induces the expression of the progesterone receptor and of *RAR*β in a subset of patients. Neither tamoxifen nor tamoxifen plus *IFN2*α potentiates the action of *ATRA* on the two genes. Once again, no significant conclusions can be drawn from the study, given the small number of patients enrolled (5 for each arm of the trial). In addition, the pharmacokinetic analyses performed in the study unveil a high level of heterogeneity among patients. Indeed, the plasma concentrations of *ATRA* determined in each patient are extremely variable and they do not show any linear correlation with the dose administered.

## Conclusions

*ATRA* is considered to be the most important and biologically active metabolite of *Vitamin-A*. In the mammalian organism, *ATRA* exerts pleiotropic effects by the transcriptional modulation of hierarchical and integrated gene-networks which are involved in the control of tissue- and cell-specific functions [10]. In the oncological field, the use of *ATRA* as a potential anti-tumor agent should keep into consideration its diverse systemic effects and its specific action on disease associated stromal and immunological cells as well as the neoplastic cells themselves. While the role of *ATRA* as a successful therapeutic agent in the treatment of *APL*, where the retinoid induces terminal differentiation of the leukemic blast, is well established, the potential of *ATRA* in the management of solid tumors is still controversial [145]. In breast cancer, the growth-inhibitory and differentiating action of *ATRA* on the neoplastic cells is documented by a large number of pre-clinical studies [5]. From a mechanistic point of view, *ATRA* controls the expression of various core components of the tumor cell dictating cell-cycle progression and the retinoid exerts a fundamental action in the stimulation of interferon-dependent responses. Its antiproliferative actions depends upon breast tumor subtype and distinct genetic characteristics (e.g., *RARA* amplification, *NOTCH1* activating deletion) [22, 23, 27]. Currently, there is little published evidence on the effects exerted by *ATRA* on the tumor micro-environment, which is an emerging determinant of the development and progression of the neoplastic disease. Indeed, in solid tumors, *ATRA* has been shown to determine opposite effects on the micro-environment, depending on the experimental setting considered. As discussed above, the retinoid seems to be effective only in a combinatorial setting where the cognate compound dictates its role. It is worth noticing that *ATRA* is a well-known and important morphogen that induces stem cell differentiation into various cell lineages and *ATRA*-based pharmacological combinations are used for the differentiation of pluripotent stem cells (*iPSC*) along different cell lineages [146, 147]. In this case too, the type of differentiation induced is dependent by the components of the combinations other than *ATRA*. With respect to the tumor niche, the action of *ATRA* on the tumor-associated immune-cell component of the micro-environment is the issue which has been the primary object of scientific attention. Of particular clinical relevance is the *ATRA* effect on the *MDSC* component sustained by a recent clinical trial [126]. However, a comprehensive picture of the role played by the retinoid is still largely incomplete. Moreover, we lack data on the action exerted by *ATRA* on other cell types of the tumor stroma, such as the adipocytes, which represent the main component of the mammary gland and are involved in retinoid storage. Thus, a full exploitation of the onco-suppressive properties of *ATRA* is likely to require further studies aimed at elucidating the action exerted by the retinoid on the tumor micro-environment. This type of studies will be facilitated by the recent development and implementation of technologies like spatial transcriptomics and spatial mass spectrometry. A final comment regards the emerging issue of drug bio-distribution in solid tumors, which suggests that investigations on new methods for the selective delivery of *ATRA* to the cancer tissue will be of fundamental importance for its efficacy.

## Data Availability

Not applicable.

## Abbreviations

ADHs: Alcohol Dehydrogenases
APL: Acute Promyelocytic Leukemia
AR: Androgen Receptor
Aregs: Adipogenesis regulators
ASPCs: Adipose Stem and Precursor Cells
ATRA: *All-trans* retinoic acid
B cells: B- lymphocytes
BRCA1: BReast CAncer gene 1
CAAs: Cancer Associated Adipocytes
CAF: Cancer Associated Fibroblasts
CAFs: Cancer Associated Fibroblasts
CEBPβ: CCAAT-Enhancer-Binding Protein beta
COUP-TFII: Chicken Ovalbumin Upstream Promoter-Transcription Factor II
CRABPI: Cytosolic Retinoic Acid Binding Protein I
CRABPII: Cytosolic Retinoic Acid Binding Protein I
CRISPR/CAS9: Clustered Regularly Interspaced Short Palindromic Repeats-Cas CRISPR-associated protein 9
CYP26A1: Cytochrome P450 Family 26 Subfamily A Member 1
CYP26B1: Cytochrome P450 Family 26 Subfamily B Member 1
CYP26C1: Cytochrome P450 Family 26 Subfamily C Member 1
DCs: Dendritic Cells
ECM: ExtraCellular Matrix
EGF: Epithelial Growth Factor
EMT: Epithelial-to-Mesenchymal Transition
ER^+^: Estrogen Receptor positive
FABP5: Fatty Acid Binding Protein 5
FGF: Fibroblast Growth Factor
GR: Glucocorticoid Receptor
HER2^+^: Human Epidermal Growth Factor Receptor 2 positive
HIF-1α: Hypoxia-Inducible Factor 1-alpha
HOXs: Homeobox genes
ICI: immune checkpoint inhibitor
IFN2α: Interferon 2α
ILCs: Innate Lymphoid Cells
iTregs: Induced regulatory T-cells
JNK2: Janus Kinase 2
LEP: Luminal EPithelial
MDSCs: Myeloid Derived Suppressor Cells
MEP: MyoEPhitelial
MMTV: Mouse Mammary Tumor Virus
MSK1: Mitogen and Stress-activated Protein Kinase-1
NK: Natural Killer
NOTCH1: Neurogenic locus NOTCH Homolog protein 1
NR1F2: Nuclear Receptor 1F2
NR2C2: Nuclear Receptor 2C2
NR2F2: Nuclear Receptor 2F2
p38MAPK: p38 Membrane Associated Protein Kinase
p42/p44 MAPK: p42/44 Membrane Associated Protein Kinase
PALB2: Partner And Localizer of BRCA2
PPARβ-δ: Peroxisome Proliferator-Activator Receptor β/δ
RALDHs: Retinaldehyde Dehydrogenases
RARE: Retinoic Acid Responsive Element
RARα: Retinoic Acid Receptor alpha
RBP4: Retinol Binding Protein 4
RDHs: Retinol Dehydrogenases
RORβ: RAR-related Orphan Receptor Beta
RXR: Retinoid X Receptor
SCC: Squamous Cell Carcinoma
SNR: Steroid Nuclear Receptors
STAT3-STAT5: Signal Transducer and Activator of Transcription 3 and 5
STRA6: Signaling Receptor And Transporter of Retinol
STRA6: Signaling Receptor And Transporter of Retinol STRA6
T cells: T- lymphocytes
TECs: Tumor-associated Endothelial Cells
TGF-β: Transforming Growth Factor-beta
TNBC: Triple Negative Breast Cancer.
TR4: Testicular Receptor 4
TTR: Transthyretin
VDR: Vitamin D Receptor
VEGF: Vascular Endothelial Growth Factor
